# Supplementary figures and images for: Pulmonary transcriptomic responses indicate a dual role of inflammation in pneumonia development and viral clearance during 2009 pandemic influenza infection
Source: PeerJ. 2017 Oct 11;5:e3915. doi: 10.7717/peerj.3915 (PMC5640978; doi:10.7717/peerj.3915)

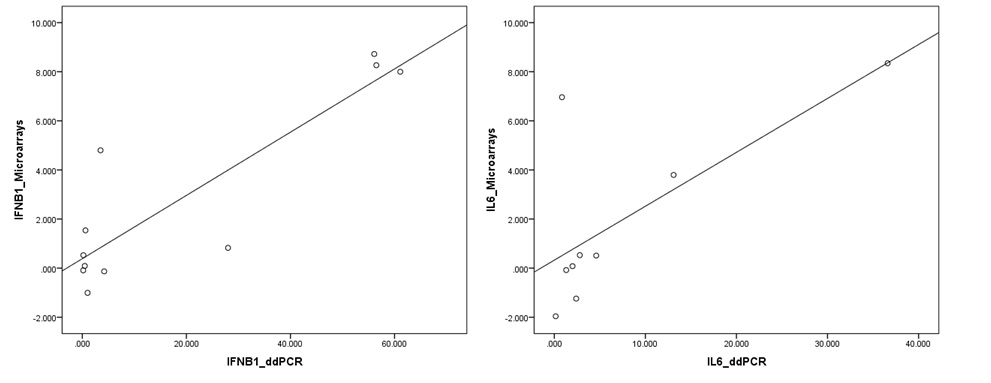

Supplement: Supplemental Information 1 — Expression values obtained from the microarrays for IFNB1 and IL6 genes showed a significant positive correlation, confirmed by using digital droplet PCR. [file peerj-05-3915-s001.jpg]

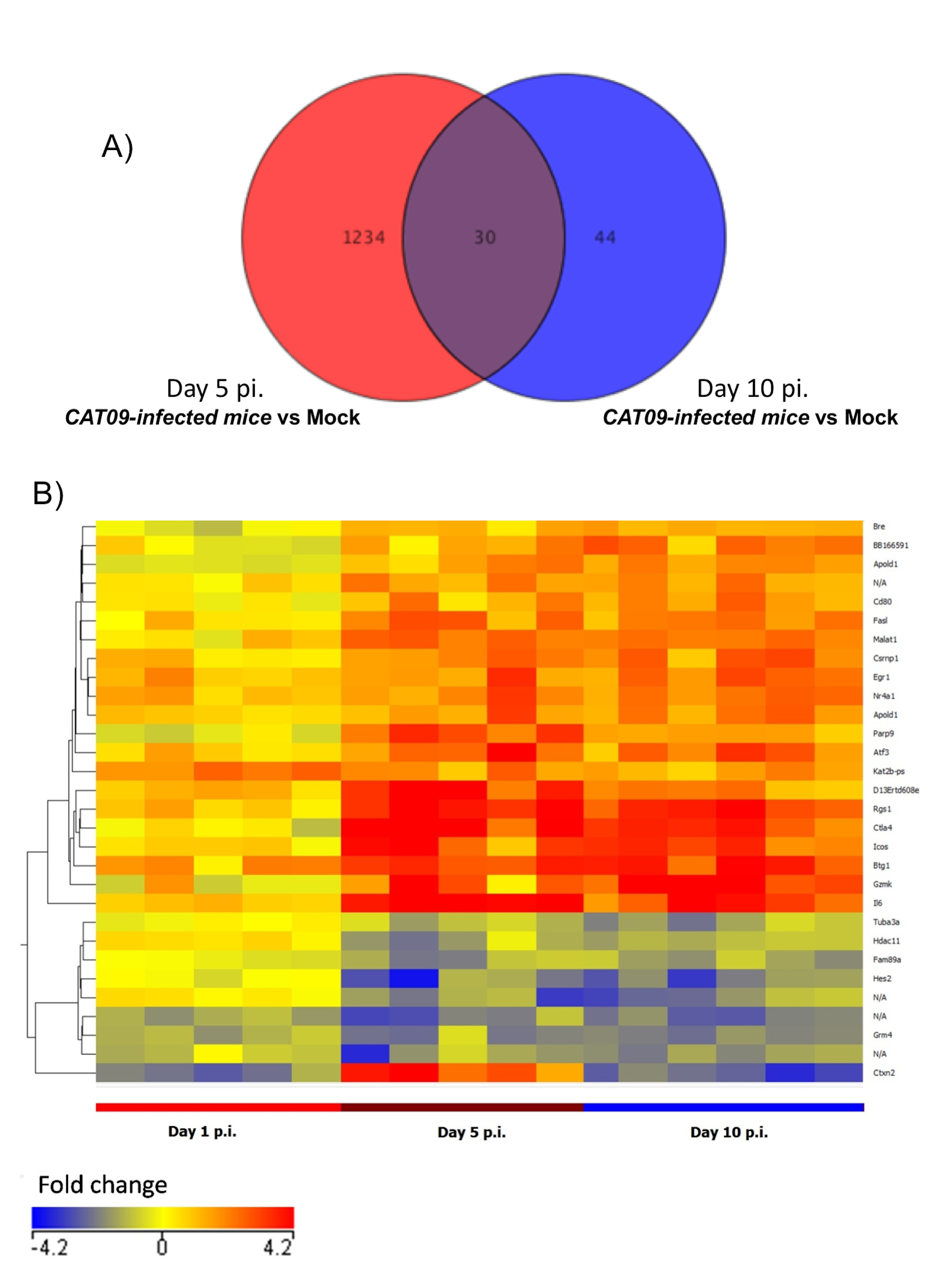

Supplement: Supplemental Information 2 — A) Venn diagram showing those genes whose expression levels differed from controls either at day 5 and day 10, and those which differed only at one time point. B) Heatmap of the common signature across different time points. The colour is proportional to their fold change (FC) compared to mock group, with the scale ranging from −4.2 FC (blue) to 4.2 FC (red). [file peerj-05-3915-s002.png]
