# Supplementary material for: Pulmonary transcriptomic responses indicate a dual role of inflammation in pneumonia development and viral clearance during 2009 pandemic influenza infection
Source: PeerJ. 2017 Oct 11;5:e3915. doi: 10.7717/peerj.3915 (PMC5640978; doi:10.7717/peerj.3915)
Supplement: Supplemental Information 4 — FC: fold change. [file peerj-05-3915-s004.doc]

***Genes involved in the top 20 canonical signalling pathways altered by A (H1N1) pdm 09 virus at day 5 post infection.***

| Ingenuity Canonical Pathways | Molecules | | | | Signaling Pathway  Categories |
| --- | --- | --- | --- | --- | --- |
| Role of Hypercytokinemia/hyperchemokinemia in the Pathogenesis of Influenza | Symbol | Entrez Gene Name | Fold Change | Type(s) | Disease-Specific Pathways; Pathogen-Influenced Signaling |
|  | CCL2 | chemokine (C-C motif) ligand 2 | 10.716 | cytokine |  |
|  | CCL4 | chemokine (C-C motif) ligand 4 | 7.854 | cytokine |  |
|  | CCR5 | chemokine (C-C motif) receptor 5 (gene/pseudogene) | 4.406 | G-protein coupled receptor |  |
|  | CXCL10 | chemokine (C-X-C motif) ligand 10 | 43.306 | cytokine |  |
|  | IFNB1 | interferon, beta 1, fibroblast | 62.582 | cytokine |  |
|  | IFNG | interferon, gamma | 12.286 | cytokine |  |
|  | IL6 | interleukin 6 | 86.593 | cytokine |  |
|  | IL12B | interleukin 12B | 4.080 | cytokine |  |
|  | IL1A | interleukin 1, alpha | 3.755 | cytokine |  |
|  | IL1B | interleukin 1, beta | 4.419 | cytokine |  |
|  | TNF | tumor necrosis factor | 4.820 | cytokine |  |
| Hepatic Fibrosis / Hepatic Stellate Cell Activation | CCL2 | chemokine (C-C motif) ligand 2 | 10.716 | cytokine | Disease-Specific Pathways; Ingenuity Toxicity List Pathways |
|  | CCR5 | chemokine (C-C motif) receptor 5 (gene/pseudogene) | 4.406 | G-protein coupled receptor |  |
|  | CXCL3 | chemokine (C-X-C motif) ligand 3 | 6.183 | cytokine |  |
|  | FASLG | Fas ligand (TNF superfamily, member 6) | 6.134 | cytokine |  |
|  | IFNG | interferon, gamma | 12.286 | cytokine |  |
|  | IGF1 | insulin-like growth factor 1 (somatomedin C) | 3.535 | growth factor |  |
|  | IGF2 | insulin-like growth factor 2 | -3.486 | growth factor |  |
|  | IL6 | interleukin 6 | 86.593 | cytokine |  |
|  | IL1A | interleukin 1, alpha | 3.755 | cytokine |  |
|  | IL1B | interleukin 1, beta | 4.419 | cytokine |  |
|  | IL1R2 | interleukin 1 receptor, type II | 4.595 | transmembrane receptor |  |
|  | KDR | kinase insert domain receptor (a type III receptor tyrosine kinase) | -6.254 | kinase |  |
|  | LEPR | leptin receptor | -5.890 | transmembrane receptor |  |
|  | LHX2 | LIM homeobox 2 | 7.958 | transcription regulator |  |
|  | MMP13 | matrix metallopeptidase 13 (collagenase 3) | 3.128 | peptidase |  |
|  | MYH9 | myosin, heavy chain 9, non-muscle | -2.973 | enzyme |  |
|  | MYH14 | myosin, heavy chain 14, non-muscle | -4.824 | other |  |
|  | MYH7B | myosin, heavy chain 7B, cardiac muscle, beta | -3.028 | other |  |
|  | PDGFB | platelet-derived growth factor beta polypeptide | -3.961 | growth factor |  |
|  | TGFB1 | transforming growth factor, beta 1 | -2.463 | growth factor |  |
|  | TIMP1 | TIMP metallopeptidase inhibitor 1 | 6.592 | cytokine |  |
|  | TNF | tumor necrosis factor | 4.820 | cytokine |  |
|  | TNFSF12 | tumor necrosis factor (ligand) superfamily, member 12 | -3.253 | cytokine |  |
|  | VEGFB | vascular endothelial growth factor B | -2.205 | growth factor |  |
| Communication between Innate and Adaptive Immune Cells | B2M | beta-2-microglobulin | 2.442 | transmembrane receptor | Cellular Immune Response |
|  | CCL4 | chemokine (C-C motif) ligand 4 | 7.854 | cytokine |  |
|  | CCL3L3 | chemokine (C-C motif) ligand 3-like 3 | 7.930 | cytokine |  |
|  | CD80 | CD80 molecule | 2.878 | transmembrane receptor |  |
|  | CD83 | CD83 molecule | 2.795 | transmembrane receptor |  |
|  | CD86 | CD86 molecule | 3.579 | transmembrane receptor |  |
|  | CXCL10 | chemokine (C-X-C motif) ligand 10 | 43.306 | cytokine |  |
|  | IFNB1 | interferon, beta 1, fibroblast | 62.582 | cytokine |  |
|  | IFNG | interferon, gamma | 12.286 | cytokine |  |
|  | IL6 | interleukin 6 | 86.593 | cytokine |  |
|  | IL12B | interleukin 12B | 4.080 | cytokine |  |
|  | IL1A | interleukin 1, alpha | 3.755 | cytokine |  |
|  | IL1B | interleukin 1, beta | 4.419 | cytokine |  |
|  | Tlr13 | toll-like receptor 13 | 2.571 | other |  |
|  | TNF | tumor necrosis factor | 4.820 | cytokine |  |
| Wnt/β-catenin Signaling | ACVR1B | activin A receptor, type IB | -3.808 | kinase | Cancer; Organismal Growth and Development |
|  | ACVR1C | activin A receptor, type IC | 5.640 | kinase |  |
|  | AKT2 | v-akt murine thymoma viral oncogene homolog 2 | -2.279 | kinase |  |
|  | APC2 | adenomatosis polyposis coli 2 | -2.268 | enzyme |  |
|  | CDH3 | cadherin 3, type 1, P-cadherin (placental) | 3.750 | other |  |
|  | CSNK1G2 | casein kinase 1, gamma 2 | -3.069 | kinase |  |
|  | DVL3 | dishevelled segment polarity protein 3 | -8.751 | other |  |
|  | FZD2 | frizzled class receptor 2 | -2.463 | G-protein coupled receptor |  |
|  | FZD7 | frizzled class receptor 7 | -4.819 | G-protein coupled receptor |  |
|  | H2BFM | H2B histone family, member M | -3.130 | other |  |
|  | LRP5 | low density lipoprotein receptor-related protein 5 | -2.690 | transmembrane receptor |  |
|  | PPP2R2C | protein phosphatase 2, regulatory subunit B, gamma | -2.681 | phosphatase |  |
|  | SOX2 | SRY (sex determining region Y)-box 2 | -3.538 | transcription regulator |  |
|  | SOX7 | SRY (sex determining region Y)-box 7 | -2.515 | transcription regulator |  |
|  | SOX13 | SRY (sex determining region Y)-box 13 | -2.444 | transcription regulator |  |
|  | SOX18 | SRY (sex determining region Y)-box 18 | -3.054 | transcription regulator |  |
|  | TGFB1 | transforming growth factor, beta 1 | -2.463 | growth factor |  |
|  | TLE1 | transducin-like enhancer of split 1 (E(sp1) homolog, Drosophila) | -2.845 | transcription regulator |  |
|  | WNT1 | wingless-type MMTV integration site family, member 1 | -8.744 | cytokine |  |
|  | WNT16 | wingless-type MMTV integration site family, member 16 | 3.664 | other |  |
|  | WNT7A | wingless-type MMTV integration site family, member 7A | -2.596 | cytokine |  |
| Agranulocyte Adhesion and Diapedesis | CCL2 | chemokine (C-C motif) ligand 2 | 10.716 | cytokine | Cellular Immune Response |
|  | Ccl2 | chemokine (C-C motif) ligand 2 | 22.767 | cytokine |  |
|  | CCL4 | chemokine (C-C motif) ligand 4 | 7.854 | cytokine |  |
|  | Ccl7 | chemokine (C-C motif) ligand 7 | 17.583 | cytokine |  |
|  | Ccl8 | chemokine (C-C motif) ligand 8 | 4.707 | other |  |
|  | CCL3L3 | chemokine (C-C motif) ligand 3-like 3 | 7.930 | cytokine |  |
|  | CLDN23 | claudin 23 | -2.553 | other |  |
|  | CXCL2 | chemokine (C-X-C motif) ligand 2 | 17.150 | cytokine |  |
|  | CXCL3 | chemokine (C-X-C motif) ligand 3 | 6.183 | cytokine |  |
|  | CXCL6 | chemokine (C-X-C motif) ligand 6 | 13.017 | cytokine |  |
|  | Cxcl9 | chemokine (C-X-C motif) ligand 9 | 21.490 | cytokine |  |
|  | CXCL10 | chemokine (C-X-C motif) ligand 10 | 43.306 | cytokine |  |
|  | CXCL13 | chemokine (C-X-C motif) ligand 13 | 9.946 | cytokine |  |
|  | IL1A | interleukin 1, alpha | 3.755 | cytokine |  |
|  | IL1B | interleukin 1, beta | 4.419 | cytokine |  |
|  | MMP13 | matrix metallopeptidase 13 (collagenase 3) | 3.128 | peptidase |  |
|  | MMP15 | matrix metallopeptidase 15 (membrane-inserted) | -3.009 | peptidase |  |
|  | MYH9 | myosin, heavy chain 9, non-muscle | -2.973 | enzyme |  |
|  | MYH14 | myosin, heavy chain 14, non-muscle | -4.824 | other |  |
|  | MYH7B | myosin, heavy chain 7B, cardiac muscle, beta | -3.028 | other |  |
|  | TNF | tumor necrosis factor | 4.820 | cytokine |  |
|  | XCL1 | chemokine (C motif) ligand 1 | 7.081 | cytokine |  |
| TREM1 Signaling | AKT2 | v-akt murine thymoma viral oncogene homolog 2 | -2.279 | kinase | Cellular Immune Response; Cytokine Signaling |
|  | CASP1 | caspase 1, apoptosis-related cysteine peptidase | 2.968 | peptidase |  |
|  | CCL2 | chemokine (C-C motif) ligand 2 | 10.716 | cytokine |  |
|  | CD83 | CD83 molecule | 2.795 | transmembrane receptor |  |
|  | CD86 | CD86 molecule | 3.579 | transmembrane receptor |  |
|  | CXCL3 | chemokine (C-X-C motif) ligand 3 | 6.183 | cytokine |  |
|  | FCGR2B | Fc fragment of IgG, low affinity IIb, receptor (CD32) | 2.974 | transmembrane receptor |  |
|  | IL6 | interleukin 6 | 86.593 | cytokine |  |
|  | IL1B | interleukin 1, beta | 4.419 | cytokine |  |
|  | IRAK1 | interleukin-1 receptor-associated kinase 1 | -2.397 | kinase |  |
|  | MAPK3 | mitogen-activated protein kinase 3 | -2.700 | kinase |  |
|  | Tlr13 | toll-like receptor 13 | 2.571 | other |  |
|  | TNF | tumor necrosis factor | 4.820 | cytokine |  |
| Differential Regulation of Cytokine Production in Intestinal Epithelial Cells by IL-17A and IL-17F | CCL2 | chemokine (C-C motif) ligand 2 | 10.716 | cytokine | Cytokine Signaling |
|  | CCL4 | chemokine (C-C motif) ligand 4 | 7.854 | cytokine |  |
|  | IFNG | interferon, gamma | 12.286 | cytokine |  |
|  | IL12B | interleukin 12B | 4.080 | cytokine |  |
|  | IL1A | interleukin 1, alpha | 3.755 | cytokine |  |
|  | IL1B | interleukin 1, beta | 4.419 | cytokine |  |
|  | TNF | tumor necrosis factor | 4.820 | cytokine |  |
| Granulocyte Adhesion and Diapedesis | CCL2 | chemokine (C-C motif) ligand 2 | 10.716 | cytokine | Cellular Immune Response |
|  | Ccl2 | chemokine (C-C motif) ligand 2 | 22.767 | cytokine |  |
|  | CCL4 | chemokine (C-C motif) ligand 4 | 7.854 | cytokine |  |
|  | Ccl7 | chemokine (C-C motif) ligand 7 | 17.583 | cytokine |  |
|  | Ccl8 | chemokine (C-C motif) ligand 8 | 4.707 | other |  |
|  | CCL3L3 | chemokine (C-C motif) ligand 3-like 3 | 7.930 | cytokine |  |
|  | CLDN23 | claudin 23 | -2.553 | other |  |
|  | CXCL2 | chemokine (C-X-C motif) ligand 2 | 17.150 | cytokine |  |
|  | CXCL3 | chemokine (C-X-C motif) ligand 3 | 6.183 | cytokine |  |
|  | CXCL6 | chemokine (C-X-C motif) ligand 6 | 13.017 | cytokine |  |
|  | Cxcl9 | chemokine (C-X-C motif) ligand 9 | 21.490 | cytokine |  |
|  | CXCL10 | chemokine (C-X-C motif) ligand 10 | 43.306 | cytokine |  |
|  | CXCL13 | chemokine (C-X-C motif) ligand 13 | 9.946 | cytokine |  |
|  | IL1A | interleukin 1, alpha | 3.755 | cytokine |  |
|  | IL1B | interleukin 1, beta | 4.419 | cytokine |  |
|  | IL1R2 | interleukin 1 receptor, type II | 4.595 | transmembrane receptor |  |
|  | MMP13 | matrix metallopeptidase 13 (collagenase 3) | 3.128 | peptidase |  |
|  | MMP15 | matrix metallopeptidase 15 (membrane-inserted) | -3.009 | peptidase |  |
|  | TNF | tumor necrosis factor | 4.820 | cytokine |  |
|  | XCL1 | chemokine (C motif) ligand 1 | 7.081 | cytokine |  |
| Altered T Cell and B Cell Signaling in Rheumatoid Arthritis | CD80 | CD80 molecule | 2.878 | transmembrane receptor | Cellular Immune Response; Disease-Specific Pathways |
|  | CD86 | CD86 molecule | 3.579 | transmembrane receptor |  |
|  | CXCL13 | chemokine (C-X-C motif) ligand 13 | 9.946 | cytokine |  |
|  | FASLG | Fas ligand (TNF superfamily, member 6) | 6.134 | cytokine |  |
|  | H2-Eb2 | histocompatibility 2, class II antigen E beta2 | 4.552 | other |  |
|  | IFNG | interferon, gamma | 12.286 | cytokine |  |
|  | IL6 | interleukin 6 | 86.593 | cytokine |  |
|  | IL12B | interleukin 12B | 4.080 | cytokine |  |
|  | IL1A | interleukin 1, alpha | 3.755 | cytokine |  |
|  | IL1B | interleukin 1, beta | 4.419 | cytokine |  |
|  | TGFB1 | transforming growth factor, beta 1 | -2.463 | growth factor |  |
|  | Tlr13 | toll-like receptor 13 | 2.571 | other |  |
|  | TNF | tumor necrosis factor | 4.820 | cytokine |  |
| Differential Regulation of Cytokine Production in Macrophages and T Helper Cells by IL-17A and IL-17F | CCL2 | chemokine (C-C motif) ligand 2 | 10.716 | cytokine | Cytokine Signaling |
|  | CCL4 | chemokine (C-C motif) ligand 4 | 7.854 | cytokine |  |
|  | IL6 | interleukin 6 | 86.593 | cytokine |  |
|  | IL12B | interleukin 12B | 4.080 | cytokine |  |
|  | IL1B | interleukin 1, beta | 4.419 | cytokine |  |
|  | TNF | tumor necrosis factor | 4.820 | cytokine |  |
| Role of IL-17F in Allergic Inflammatory Airway Diseases | CCL2 | chemokine (C-C motif) ligand 2 | 10.716 | cytokine | Cytokine Signaling; Disease-Specific Pathways |
|  | CCL4 | chemokine (C-C motif) ligand 4 | 7.854 | cytokine |  |
|  | CXCL6 | chemokine (C-X-C motif) ligand 6 | 13.017 | cytokine |  |
|  | CXCL10 | chemokine (C-X-C motif) ligand 10 | 43.306 | cytokine |  |
|  | IGF1 | insulin-like growth factor 1 (somatomedin C) | 3.535 | growth factor |  |
|  | IL6 | interleukin 6 | 86.593 | cytokine |  |
|  | IL1B | interleukin 1, beta | 4.419 | cytokine |  |
|  | MAPK3 | mitogen-activated protein kinase 3 | -2.700 | kinase |  |
|  | MMP13 | matrix metallopeptidase 13 (collagenase 3) | 3.128 | peptidase |  |
| Crosstalk between Dendritic Cells and Natural Killer Cells | CAMK2A | calcium/calmodulin-dependent protein kinase II alpha | -2.895 | kinase | Cellular Immune Response |
|  | CD69 | CD69 molecule | 7.814 | transmembrane receptor |  |
|  | CD80 | CD80 molecule | 2.878 | transmembrane receptor |  |
|  | CD83 | CD83 molecule | 2.795 | transmembrane receptor |  |
|  | CD86 | CD86 molecule | 3.579 | transmembrane receptor |  |
|  | FASLG | Fas ligand (TNF superfamily, member 6) | 6.134 | cytokine |  |
|  | FSCN3 | fascin actin-bundling protein 3, testicular | 6.451 | other |  |
|  | IFNB1 | interferon, beta 1, fibroblast | 62.582 | cytokine |  |
|  | IFNG | interferon, gamma | 12.286 | cytokine |  |
|  | IL6 | interleukin 6 | 86.593 | cytokine |  |
|  | IL12B | interleukin 12B | 4.080 | cytokine |  |
|  | KLRD1 | killer cell lectin-like receptor subfamily D, member 1 | 3.451 | transmembrane receptor |  |
|  | TNF | tumor necrosis factor | 4.820 | cytokine |  |
| HMGB1 Signaling | AGER | advanced glycosylation end product-specific receptor | -2.557 | transmembrane receptor | Cellular Immune Response; Cellular Stress and Injury; Cytokine Signaling; Humoral Immune Response |
|  | AKT2 | v-akt murine thymoma viral oncogene homolog 2 | -2.279 | kinase |  |
|  | CCL2 | chemokine (C-C motif) ligand 2 | 10.716 | cytokine |  |
|  | IFNG | interferon, gamma | 12.286 | cytokine |  |
|  | IL6 | interleukin 6 | 86.593 | cytokine |  |
|  | IL12B | interleukin 12B | 4.080 | cytokine |  |
|  | IL1A | interleukin 1, alpha | 3.755 | cytokine |  |
|  | IL1B | interleukin 1, beta | 4.419 | cytokine |  |
|  | MAPK3 | mitogen-activated protein kinase 3 | -2.700 | kinase |  |
|  | RHOA | ras homolog family member A | -2.800 | enzyme |  |
|  | RHOV | ras homolog family member V | -2.815 | enzyme |  |
|  | RND2 | Rho family GTPase 2 | -2.791 | enzyme |  |
|  | RND3 | Rho family GTPase 3 | 2.624 | enzyme |  |
|  | TGFB1 | transforming growth factor, beta 1 | -2.463 | growth factor |  |
|  | TNF | tumor necrosis factor | 4.820 | cytokine |  |
| Graft-versus-Host Disease Signaling | CD80 | CD80 molecule | 2.878 | transmembrane receptor | Cellular Immune Response; Disease-Specific Pathways |
|  | CD86 | CD86 molecule | 3.579 | transmembrane receptor |  |
|  | FASLG | Fas ligand (TNF superfamily, member 6) | 6.134 | cytokine |  |
|  | H2-Eb2 | histocompatibility 2, class II antigen E beta2 | 4.552 | other |  |
|  | IFNG | interferon, gamma | 12.286 | cytokine |  |
|  | IL6 | interleukin 6 | 86.593 | cytokine |  |
|  | IL1A | interleukin 1, alpha | 3.755 | cytokine |  |
|  | IL1B | interleukin 1, beta | 4.419 | cytokine |  |
|  | TNF | tumor necrosis factor | 4.820 | cytokine |  |
| T Helper Cell Differentiation | CD80 | CD80 molecule | 2.878 | transmembrane receptor | Cellular Immune Response; Cytokine Signaling |
|  | CD86 | CD86 molecule | 3.579 | transmembrane receptor |  |
|  | H2-Eb2 | histocompatibility 2, class II antigen E beta2 | 4.552 | other |  |
|  | ICOS | inducible T-cell co-stimulator | 7.637 | transmembrane receptor |  |
|  | IFNG | interferon, gamma | 12.286 | cytokine |  |
|  | IL6 | interleukin 6 | 86.593 | cytokine |  |
|  | IL12B | interleukin 12B | 4.080 | cytokine |  |
|  | IL12RB1 | interleukin 12 receptor, beta 1 | 4.396 | transmembrane receptor |  |
|  | IL12RB2 | interleukin 12 receptor, beta 2 | -3.570 | transmembrane receptor |  |
|  | TGFB1 | transforming growth factor, beta 1 | -2.463 | growth factor |  |
|  | TNF | tumor necrosis factor | 4.820 | cytokine |  |
| Atherosclerosis Signaling | APOA2 | apolipoprotein A-II | -2.420 | transporter | Cardiovascular Signaling; Disease-Specific Pathways |
|  | APOD | apolipoprotein D | 4.580 | transporter |  |
|  | CCL2 | chemokine (C-C motif) ligand 2 | 10.716 | cytokine |  |
|  | IFNG | interferon, gamma | 12.286 | cytokine |  |
|  | IL6 | interleukin 6 | 86.593 | cytokine |  |
|  | IL1A | interleukin 1, alpha | 3.755 | cytokine |  |
|  | IL1B | interleukin 1, beta | 4.419 | cytokine |  |
|  | MMP13 | matrix metallopeptidase 13 (collagenase 3) | 3.128 | peptidase |  |
|  | MSR1 | macrophage scavenger receptor 1 | 4.698 | transmembrane receptor |  |
|  | PDGFB | platelet-derived growth factor beta polypeptide | -3.961 | growth factor |  |
|  | PLA2G7 | phospholipase A2, group VII (platelet-activating factor acetylhydrolase, plasma) | 2.817 | enzyme |  |
|  | TGFB1 | transforming growth factor, beta 1 | -2.463 | growth factor |  |
|  | TNF | tumor necrosis factor | 4.820 | cytokine |  |
|  | TNFSF12 | tumor necrosis factor (ligand) superfamily, member 12 | -3.253 | cytokine |  |
|  | TPSAB1/TPSB2 | tryptase alpha/beta 1 | 7.113 | peptidase |  |
| Role of Macrophages, Fibroblasts and Endothelial Cells in Rheumatoid Arthritis | ADAMTS4 | ADAM metallopeptidase with thrombospondin type 1 motif, 4 | 6.710 | peptidase | Disease-Specific Pathways |
|  | AKT2 | v-akt murine thymoma viral oncogene homolog 2 | -2.279 | kinase |  |
|  | APC2 | adenomatosis polyposis coli 2 | -2.268 | enzyme |  |
|  | CAMK2A | calcium/calmodulin-dependent protein kinase II alpha | -2.895 | kinase |  |
|  | CCL2 | chemokine (C-C motif) ligand 2 | 10.716 | cytokine |  |
|  | CEBPD | CCAAT/enhancer binding protein (C/EBP), delta | 2.847 | transcription regulator |  |
|  | FZD2 | frizzled class receptor 2 | -2.463 | G-protein coupled receptor |  |
|  | FZD7 | frizzled class receptor 7 | -4.819 | G-protein coupled receptor |  |
|  | IL6 | interleukin 6 | 86.593 | cytokine |  |
|  | IL1A | interleukin 1, alpha | 3.755 | cytokine |  |
|  | IL1B | interleukin 1, beta | 4.419 | cytokine |  |
|  | IL1R2 | interleukin 1 receptor, type II | 4.595 | transmembrane receptor |  |
|  | IRAK1 | interleukin-1 receptor-associated kinase 1 | -2.397 | kinase |  |
|  | LRP5 | low density lipoprotein receptor-related protein 5 | -2.690 | transmembrane receptor |  |
|  | MAPK3 | mitogen-activated protein kinase 3 | -2.700 | kinase |  |
|  | MMP13 | matrix metallopeptidase 13 (collagenase 3) | 3.128 | peptidase |  |
|  | PDGFB | platelet-derived growth factor beta polypeptide | -3.961 | growth factor |  |
|  | RHOA | ras homolog family member A | -2.800 | enzyme |  |
|  | SOCS1 | suppressor of cytokine signaling 1 | 6.446 | other |  |
|  | TGFB1 | transforming growth factor, beta 1 | -2.463 | growth factor |  |
|  | Tlr13 | toll-like receptor 13 | 2.571 | other |  |
|  | TNF | tumor necrosis factor | 4.820 | cytokine |  |
|  | VEGFB | vascular endothelial growth factor B | -2.205 | growth factor |  |
|  | WNT1 | wingless-type MMTV integration site family, member 1 | -8.744 | cytokine |  |
|  | WNT16 | wingless-type MMTV integration site family, member 16 | 3.664 | other |  |
|  | WNT7A | wingless-type MMTV integration site family, member 7A | -2.596 | cytokine |  |
| Colorectal Cancer Metastasis Signaling | AKT2 | v-akt murine thymoma viral oncogene homolog 2 | -2.279 | kinase | Cancer; Disease-Specific Pathways |
|  | FZD2 | frizzled class receptor 2 | -2.463 | G-protein coupled receptor |  |
|  | FZD7 | frizzled class receptor 7 | -4.819 | G-protein coupled receptor |  |
|  | IFNG | interferon, gamma | 12.286 | cytokine |  |
|  | IL6 | interleukin 6 | 86.593 | cytokine |  |
|  | LRP5 | low density lipoprotein receptor-related protein 5 | -2.690 | transmembrane receptor |  |
|  | MAPK3 | mitogen-activated protein kinase 3 | -2.700 | kinase |  |
|  | MMP13 | matrix metallopeptidase 13 (collagenase 3) | 3.128 | peptidase |  |
|  | MMP15 | matrix metallopeptidase 15 (membrane-inserted) | -3.009 | peptidase |  |
|  | PRKACA | protein kinase, cAMP-dependent, catalytic, alpha | -2.662 | kinase |  |
|  | PTGS2 | prostaglandin-endoperoxide synthase 2 (prostaglandin G/H synthase and cyclooxygenase) | 3.454 | enzyme |  |
|  | RHOA | ras homolog family member A | -2.800 | enzyme |  |
|  | RHOV | ras homolog family member V | -2.815 | enzyme |  |
|  | RND2 | Rho family GTPase 2 | -2.791 | enzyme |  |
|  | RND3 | Rho family GTPase 3 | 2.624 | enzyme |  |
|  | TGFB1 | transforming growth factor, beta 1 | -2.463 | growth factor |  |
|  | Tlr13 | toll-like receptor 13 | 2.571 | other |  |
|  | TNF | tumor necrosis factor | 4.820 | cytokine |  |
|  | VEGFB | vascular endothelial growth factor B | -2.205 | growth factor |  |
|  | WNT1 | wingless-type MMTV integration site family, member 1 | -8.744 | cytokine |  |
|  | WNT16 | wingless-type MMTV integration site family, member 16 | 3.664 | other |  |
|  | WNT7A | wingless-type MMTV integration site family, member 7A | -2.596 | cytokine |  |
| Role of Osteoblasts, Osteoclasts and Chondrocytes in Rheumatoid Arthritis | CASP1 | caspase 1, apoptosis-related cysteine peptidase | 2.968 | peptidase | Disease-Specific Pathways |
|  | IFIH1 | interferon induced with helicase C domain 1 | 2.624 | enzyme |  |
|  | IFNB1 | interferon, beta 1, fibroblast | 62.582 | cytokine |  |
|  | IFNG | interferon, gamma | 12.286 | cytokine |  |
|  | IL6 | interleukin 6 | 86.593 | cytokine |  |
|  | IL12B | interleukin 12B | 4.080 | cytokine |  |
|  | IL1A | interleukin 1, alpha | 3.755 | cytokine |  |
|  | IL1B | interleukin 1, beta | 4.419 | cytokine |  |
|  | MAPK3 | mitogen-activated protein kinase 3 | -2.700 | kinase |  |
|  | OAS1 | 2'-5'-oligoadenylate synthetase 1, 40/46kDa | 6.447 | enzyme |  |
|  | OAS2 | 2'-5'-oligoadenylate synthetase 2, 69/71kDa | 6.317 | enzyme |  |
|  | Oas1f | 2'-5' oligoadenylate synthetase 1F | 5.202 | other |  |
|  | TGFB1 | transforming growth factor, beta 1 | -2.463 | growth factor |  |
|  | TNF | tumor necrosis factor | 4.820 | cytokine |  |
| Role of Pattern Recognition Receptors in Recognition of Bacteria and Viruses | DLL3 | delta-like 3 (Drosophila) | -3.853 | other | Cellular Immune Response; Pathogen-Influenced Signaling |
|  | HES1 | hes family bHLH transcription factor 1 | -3.085 | transcription regulator |  |
|  | HES7 | hes family bHLH transcription factor 7 | -2.860 | transcription regulator |  |
|  | LFNG | LFNG O-fucosylpeptide 3-beta-N-acetylglucosaminyltransferase | -3.054 | enzyme |  |
|  | NCSTN | nicastrin | -2.766 | peptidase |  |
|  | NOTCH3 | notch 3 | -2.175 | transcription regulator |  |
|  | RFNG | RFNG O-fucosylpeptide 3-beta-N-acetylglucosaminyltransferase | -2.662 | enzyme |  |
