# Supplementary material for: Pulmonary transcriptomic responses indicate a dual role of inflammation in pneumonia development and viral clearance during 2009 pandemic influenza infection
Source: PeerJ. 2017 Oct 11;5:e3915. doi: 10.7717/peerj.3915 (PMC5640978; doi:10.7717/peerj.3915)
Supplement: Supplemental Information 6 — This table summarized the most significant previous studies evaluating the transcriptomic response to A (H1N1) pdm09 virus in different animal models. EID50: 50% Egg Infective Dose, TCID50: 50% Tissue Culture Infective Dose, PFU: Plaque Forming Unit, dpi: days post infection. [file peerj-05-3915-s006.doc]

**Studies evaluating host transcriptomic responses to A (H1N1) pdm09** influenza virus in animal models

| **Reference** | **Animal model** | **A (H1N1) pmd09 virus strain** | **A (H1N1) pdm09 virus dose** | **Transcriptomic analysis** | **Tissue** | **Time points (dpi)** | **Scope** |
| --- | --- | --- | --- | --- | --- | --- | --- |
| (Rowe et al., 2010) | Ferret | A/California/07/2009 | 10^6^ EID_50_ | Microarrays | Lungs | 1, 3, 5, 7 and 14 | Understand A (H1N1) pmd09 induced pathogenesis and the evolution of host immune responses |
| (Ma et al., 2011) | Swine | A/CA/04/2009 (human), A/swine/Alberta/25/2009 | 10^6^ TCID_50_ | Microarrays | Lungs | 3 and 5 | Compare the pathogenesis of two A (H1N1) pmd09 viruses with that of the 1918-like classical swine influenza virus |
| (Camp et al., 2012) | Ferret | A/California/07/2009 | 10^4^ TCID_50_/ml | RNA Sequencing | Lung, heart, bone marrow from the femur, lymph node, spleen and blood. | 3 and 6 | Enable de novo identification of transcriptionally active ferret genes in response to infection |
| (Ljungberg et al., 2012) | Ferret | A/California/07/2009, A/Mexico/4482/2009 | 10^6^ TCID_50_/ml | Microarrays | Blood | 1, 2, 3, 5 and 7 | Identify different host gene responses based on both strain (A (H1N1) pmd09 and seasonal H1N1 influenza virus) and severity of disease. |
| (Josset et al., 2012b) | Macaque | A/California/04/2009 | 7 x 10^6^ TCID_50_ | Microarrays | BAL fluid cells | 0,4,7,10 and 14 | Characterize the impact of age on the host response to A (H1N1) pmd09 infection in the absence of pre-existing immunity |
| (Josset et al., 2012a) | Mouse | A/California/04/2009, mouse-adapted MA1-A/ California/04/2009 and A/Mexico/4482/2009 | 10^6^ PFU | Microarrays | Lungs | 1,3 and 5 | Characterize the differences in the host response to prototypic A (H1N1) pmd09 strain and a corresponding mouse-adapted variant |
| (Go et al., 2012) | Mouse, swine and macaque | A/California/04/2009 | 10^6^ PFU | Microarrays | Lungs | 1,3 and 5 dpi | Characterize host responses to A (H1N1) pmd09 virus during acute infection across multiple model systems |
| (León et al., 2013) | Ferret | A/California/07/2009 | 10^6^ EID_50_ | Microarrays and RNA sequencing | Lungs and lymph nodes | 0,3,5,7 and 14 | Characterize the infectome during A (H1N1) pmd09 infection in experimentally infected ferrets |
| (Tchitchek et al., 2013) | Mouse | Recombinant A/California/04/2009 | 10^2^, 10^3^, 10^4^, 10^5^ or 10^6^ PFU | Microarrays | Lungs | 1,2,4 and 7 | Studying molecular dynamics of the host response and the complex interplay between virus-encoded determinants, host regulatory factors, H5N1 pathogenesis and severe lung disease |
| (Zou et al., 2013) | Mouse | Swine (Jia6/10) and human (LN/09)A H1N1/2009 viruses. | 10^3^ TCID_50_ | RNA Sequencing | Lungs | 3 and 5 | Investigate the different pathogenesis and the mechanism involving in the interaction between host and swine- and human-origin A (H1N1) pmd09 viruses |
| (Morrison et al., 2014) | Mouse | A/Mexico/4482/2007 | 10^5^ PFU | microarrays | Lungs | 1,3 and 5 | Characterize the host transcriptomic response to emerging H7N9 influenza virus and compare it with the responses to H7N7, H5N1, and A (H1N1) pmd09. |
| (Lin et al., 2015) | Swine | A/swine/Hubei/101/2009 | 10^7^ EID_50_ | microarrays | Lungs | 3 and 7 | Explore global host response induced by A (H1N1) pmd09 swine influenza virus in pigs |
| (Shoemaker et al., 2015) | Mouse | A/California/04/2009 A/Kawasaki/UTK-4/2009 | 10^5^ PFU | microarrays | Lungs | 1, 3, 5 and 7 | Create mathematical models of the host inflammatory response induced by virulent influenza strains |
| (Peng et al., 2014) | Ferret | A/California/04/2009 | 10^6^ PFU | RNA Sequencing | Lungs | 1, 3 and 8 | Characterize the ferret host response to two H1N1 influenza viruses [A (H1N1) pmd09 and 1918 pandemic influenza viruses) by RNA-seq |
| (Manchanda et al., 2016) | Mouse | once mouse lung-passaged variant mpJena/5258 | 10^6^ TCID_50_ | microarrays | Lungs | 1, 2, 3, 4, 5, 6, 7, 9, and 12 | Studying the dynamics of intra-host evolution of A (H1N1) pmd09 virus |

**References:**

Camp JV., Svensson TL., McBrayer A., Jonsson CB., Liljeström P., Bruder CE. 2012. De-novo transcriptome sequencing of a normalized cDNA pool from influenza infected ferrets. *PloS One* 7:e37104. DOI: 10.1371/journal.pone.0037104.

Go JT., Belisle SE., Tchitchek N., Tumpey TM., Ma W., Richt JA., Safronetz D., Feldmann H., Katze MG. 2012. 2009 pandemic H1N1 influenza virus elicits similar clinical course but differential host transcriptional response in mouse, macaque, and swine infection models. *BMC genomics* 13:627. DOI: 10.1186/1471-2164-13-627.

Josset L., Belser JA., Pantin-Jackwood MJ., Chang JH., Chang ST., Belisle SE., Tumpey TM., Katze MG. 2012a. Implication of inflammatory macrophages, nuclear receptors, and interferon regulatory factors in increased virulence of pandemic 2009 H1N1 influenza A virus after host adaptation. *Journal of Virology* 86:7192–7206. DOI: 10.1128/JVI.00563-12.

Josset L., Engelmann F., Haberthur K., Kelly S., Park B., Kawoaka Y., García-Sastre A., Katze MG., Messaoudi I. 2012b. Increased viral loads and exacerbated innate host responses in aged macaques infected with the 2009 pandemic H1N1 influenza A virus. *Journal of Virology* 86:11115–11127. DOI: 10.1128/JVI.01571-12.

León AJ., Banner D., Xu L., Ran L., Peng Z., Yi K., Chen C., Xu F., Huang J., Zhao Z., Lin Z., Huang SHS., Fang Y., Kelvin AA., Ross TM., Farooqui A., Kelvin DJ. 2013. Sequencing, annotation, and characterization of the influenza ferret infectome. *Journal of Virology* 87:1957–1966. DOI: 10.1128/JVI.02476-12.

Lin X., Huang C., Shi J., Wang R., Sun X., Liu X., Zhao L., Jin M. 2015. Investigation of Pathogenesis of H1N1 Influenza Virus and Swine Streptococcus suis Serotype 2 Co-Infection in Pigs by Microarray Analysis. *PloS One* 10:e0124086. DOI: 10.1371/journal.pone.0124086.

Ljungberg K., McBrayer A., Camp JV., Chu Y-K., Tapp R., Noah DL., Grimes S., Proctor ML., Liljeström P., Jonsson CB., Bruder CE. 2012. Host gene expression signatures discriminate between ferrets infected with genetically similar H1N1 strains. *PloS One* 7:e40743. DOI: 10.1371/journal.pone.0040743.

Ma W., Belisle SE., Mosier D., Li X., Stigger-Rosser E., Liu Q., Qiao C., Elder J., Webby R., Katze MG., Richt JA. 2011. 2009 pandemic H1N1 influenza virus causes disease and upregulation of genes related to inflammatory and immune responses, cell death, and lipid metabolism in pigs. *Journal of Virology* 85:11626–11637. DOI: 10.1128/JVI.05705-11.

Manchanda H., Seidel N., Blaess MF., Claus RA., Linde J., Slevogt H., Sauerbrei A., Guthke R., Schmidtke M. 2016. Differential Biphasic Transcriptional Host Response Associated with Coevolution of Hemagglutinin Quasispecies of Influenza A Virus. *Frontiers in Microbiology* 7:1167. DOI: 10.3389/fmicb.2016.01167.

Morrison J., Josset L., Tchitchek N., Chang J., Belser JA., Swayne DE., Pantin-Jackwood MJ., Tumpey TM., Katze MG. 2014. H7N9 and other pathogenic avian influenza viruses elicit a three-pronged transcriptomic signature that is reminiscent of 1918 influenza virus and is associated with lethal outcome in mice. *Journal of Virology* 88:10556–10568. DOI: 10.1128/JVI.00570-14.

Peng X., Alföldi J., Gori K., Eisfeld AJ., Tyler SR., Tisoncik-Go J., Brawand D., Law GL., Skunca N., Hatta M., Gasper DJ., Kelly SM., Chang J., Thomas MJ., Johnson J., Berlin AM., Lara M., Russell P., Swofford R., Turner-Maier J., Young S., Hourlier T., Aken B., Searle S., Sun X., Yi Y., Suresh M., Tumpey TM., Siepel A., Wisely SM., Dessimoz C., Kawaoka Y., Birren BW., Lindblad-Toh K., Di Palma F., Engelhardt JF., Palermo RE., Katze MG. 2014. The draft genome sequence of the ferret (Mustela putorius furo) facilitates study of human respiratory disease. *Nature Biotechnology* 32:1250–1255. DOI: 10.1038/nbt.3079.

Rowe T., León AJ., Crevar CJ., Carter DM., Xu L., Ran L., Fang Y., Cameron CM., Cameron MJ., Banner D., Ng DC., Ran R., Weirback HK., Wiley CA., Kelvin DJ., Ross TM. 2010. Modeling host responses in ferrets during A/California/07/2009 influenza infection. *Virology* 401:257–265. DOI: 10.1016/j.virol.2010.02.020.

Shoemaker JE., Fukuyama S., Eisfeld AJ., Zhao D., Kawakami E., Sakabe S., Maemura T., Gorai T., Katsura H., Muramoto Y., Watanabe S., Watanabe T., Fuji K., Matsuoka Y., Kitano H., Kawaoka Y. 2015. An Ultrasensitive Mechanism Regulates Influenza Virus-Induced Inflammation. *PLoS pathogens* 11:e1004856. DOI: 10.1371/journal.ppat.1004856.

Tchitchek N., Eisfeld AJ., Tisoncik-Go J., Josset L., Gralinski LE., Bécavin C., Tilton SC., Webb-Robertson B-J., Ferris MT., Totura AL., Li C., Neumann G., Metz TO., Smith RD., Waters KM., Baric R., Kawaoka Y., Katze MG. 2013. Specific mutations in H5N1 mainly impact the magnitude and velocity of the host response in mice. *BMC systems biology* 7:69. DOI: 10.1186/1752-0509-7-69.

Zou W., Chen D., Xiong M., Zhu J., Lin X., Wang L., Zhang J., Chen L., Zhang H., Chen H., Chen M., Jin M. 2013. Insights into the increasing virulence of the swine-origin pandemic H1N1/2009 influenza virus. *Scientific Reports* 3:1601. DOI: 10.1038/srep01601.
